# Mass drug administration for trachoma elimination in a pastoral conflict area in Baringo County, Kenya: A study on barriers to coverage and compliance

**DOI:** 10.1371/journal.pntd.0013327

**Published:** 2025-08-04

**Authors:** Bridget W. Kimani, Tabitha W. Kanyui, Paul M. Gichuki, Collins O. Okoyo, Titus K. Watitu, Wyckliff P. Omondi, Doris W. Njomo

**Affiliations:** 1 Eastern and Southern Africa Centre of Infectious Parasite Control, Kenya Medical Research Institute (KEMRI), Nairobi, Kenya; 2 National Trachoma Programme Manager, Ministry of Health, Nairobi, Kenya; 3 Head Division of Vector Borne and Neglected Tropical Diseases (DVBNTDs), Ministry of Health, Nairobi, Kenya; National Institutes of Health, UNITED STATES OF AMERICA

## Abstract

Trachoma, a neglected tropical disease (NTD), is the leading infectious cause of blindness worldwide. It is caused by repeated ocular infections with the bacteria *Chlamydia trachomatis*. Treatment coverage achieved in Baringo County, Kenya in 2020 and 2021 was 80% and 79% respectively, further investigation at the lower levels known as wards shows coverages ranging between 48% to 57% which are far below the WHO recommended threshold of at least 80% of the target population should be reached with MDA The objective of this study was to identify barriers of community participation and access to mass drug administration for trachoma elimination in a pastoral conflict area of Baringo County, Kenya. Sixteen focus group discussions were conducted with community members and four among community health volunteers. Eight county and sub-county leaders and fourteen opinion leaders participated in in-depth interviews. Patients with trichiasis took part in six micro-narrative surveys. All study participants were purposively selected and the number of FGDs, interviews, and micro-narrative surveys were determined by the saturation model, an iterative design was employed for data collection and analysis and further sample selection to give early insights and influence the selection of more participants. Although community members were aware of trachoma and the benefits of MDA, poor knowledge of trachoma etiology exists, with misconceptions attributing it to dirt, dust, flies, and even witchcraft. Women, children, and elderly persons were perceived to be at a higher risk of infection. The remoteness of some villages and towns hindered awareness creation resulting in some community members being unaware of trachoma and MDA. Side effects like diarrhea, nausea, headache, and drowsiness discourage participation. Drug size and taste are perceived as negative factors. Lack of information on side effects and their management contributes to hesitancy. Considering the perspectives of nomadic people and understanding context-specific risks, supporting the design of community-led interventions is critical in the development of effective MDA strategies, which can aid in halting trachoma transmission.

## Author summary

Trachoma is a contagious infection of the eye and the leading cause of blindness worldwide. The neglected tropical disease road map 2021–2030, set its new target for global elimination of trachoma from 2020 to 2030. Despite significant progress in fighting trachoma in Kenya, challenges persist, evident in the prevalence of the disease among nomadic communities and the Maasai cattle corridors. These communities engage in frequent migration, which poses unique obstacles for disease control due to the need to optimize scarce resources, especially pasture and water. MDA has been widely applied to eliminate or interrupt the transmission of trachoma in endemic regions without requiring individual diagnosis. It is safe, effective and inexpensive. This qualitative study aimed at assessing contextual, social and environmental barriers to participation and access to MDA for trachoma elimination.

## Introduction

Trachoma, an ocular infection caused by Chlamydia trachomatis, is a neglected tropical disease (NTD) found predominantly in developing countries. It is the leading infectious cause of blindness and is endemic in 53 countries. Active infection often begins during infancy or childhood and can become chronic. The bacteria are spread by direct contact with eye and nose discharges from infected individuals, by interaction with fomites (i.e., inanimate objects that carry infectious agents, such as towels or washcloths), or by eye-seeking flies (particularly Musca sorbens). An estimated 325 million people live in areas where they can be exposed to trachoma, and more than 7 million suffer from trichiasis, the final painful stage of this eye disease [1,2].

The World Health Organization (WHO) estimates that 137 million people worldwide are infected with trachoma, thus making it a leading cause of infectious blindness globally and a public health priority. Currently, the disease is still endemic in 44 countries [2,3]. The Surgery, Antibiotic, [3] Facial cleanliness and Environmental hygiene (SAFE) strategy was developed to eliminate trachoma by 2030 [2]. Surgery helps in the prevention of pain and related symptoms, the worsening of trichiasis, and the preservation of visual acuity [4]. Delivery of antibiotics through MDA is effective in reducing the prevalence of active trachoma, preventing disease transmission, thus reducing poverty among the poorest sectors of communities [3]. Facial (F) cleanliness and environmental (E) hygiene components largely depend on behavioural change interventions in schools and communities [5].

In Kenya, Trachoma is endemic in 12 of the 47 counties where a total population of approximately 11 million people live [6]. Out of the 12 counties, 8 are still active with Trachomatous Follicular [7]. More than 53,200 Kenyans have already been blinded by trachoma and an estimated 370,000 children are currently living with active infections. The trachoma MDA programme in Kenya is implemented by the Ministry of Health, with support from partners such as the Fred Hollows Foundation, and Operation Eyesight Universal. All members of the community are targeted for treatment during the MDA campaign as per the Programme dosing guidelines [8].

Treatment coverage achieved in Baringo County, Kenya in 2020 and 2021 was 80% and 79% respectively, further investigation at the lower levels known as wards shows coverages ranging between 48% to 57% which are far below the WHO recommended threshold of at least 80%. of the target population should be reached with MDA [9]. Using community participation and action there is a need to identify barriers that could be a hindrance to the achievement of high treatment uptake, identify opportunities, and develop strategies aimed at addressing such barriers. This study aimed to address barriers of community participation and access to mass drug administration for trachoma elimination using participatory approaches in a pastoral conflict area of Baringo County, Kenya. This was achieved through the use of various qualitative data collection techniques that included focus group discussions, in-depth interviews, and micronarrative surveys.

## Materials and methods

### Ethics statement

Ethical clearance was received from the Kenya Medical Research Institute (KEMRI), Scientific and Ethics Review Unit (SERU No. 4532). Permission to carry out the study was obtained from the Kenya National Commission for Science and Technology and the respective authorities of Baringo County. Written informed consent was sought from all the study participants. An information sheet was provided to all individuals who were 18 years and above invited to participate in the study in *Pokomo*, the local language. During data capture and transcription, participant names were replaced with alphanumeric unique identifiers to ensure anonymity and confidentiality.

### Study area

Baringo County is made up of seven sub-counties including Tiaty East [10]. It is classified under Arid and Semi-arid Lands (ASAL). It is located in the eastern part of the former East Pokot district, in the Lake Baringo basin adjacent to the eastern highlands near the Laikipia plateau in Kenya’s Central Rift Valley. Tiaty East occupies 2163 km2 with a population of approximately 73,400 people [11]. It displays a rugged topography of lowland plains, rolling hills, and mountain ranges, dominated by semi-arid and acacia bush savannah. Rainfall is characterized by high inter-annual variation following a bi-modal pattern [12]. There is a total of 172 health facilities; seven level 4, 25 level 3, and 140 level 2 centers [13]. Tiaty East sub-county is composed of 4 wards, that is, Silale, Loyamorok, Tangulbei, and Churo-Amaya [10].

The inhabitants are almost exclusively Nilotic-speaking Pokot people whose dominant form of land use is pastoralism in almost the entire area over the past 200 years [14]. The road network in Tiaty East consists of two main roads, the main north-south road that connects Tiaty East with the city of Nakuru, and the main east-west road connecting Loruk with Laikipia County. In Tiaty East walking is the main form of transportation [12]. Livestock is the main export from the area. It is often driven through the bush to one of the main markets in Ng’inyang or Amaya [12]. Infrequent bus connections to and from Marigat form the main means of public transportation, the small town serves as a transportation hub to other parts of the region [15]. Recent investments include the setting up of large-scale geothermal energy stations [16].

Conflicts often revolve around territorial boundaries and (grazing) lands, involving mutual livestock raiding and armed assaults [17,18]. These clashes are often deadly as they revolve around large-scale attacks and counter-attacks involving police and army operations, as well as the ambushing and killing of state security personnel. The entire population is usually held responsible for insurgent activities by state forces who have not been squeamish about collective punishments, such as random destruction of houses, beatings, and livestock confiscation [19]. As a result, many households chose to settle hidden in the bush and at a distance from roads, particularly those with more livestock. Most of the old colonial roads are no longer traversable by car and are only used by pedestrians [20]. For this study, the Loyamorok ward was purposively selected owing to its low treatment coverage trends, nomadic lifestyle, intercounty cross-border movement, and conflicts. All the nine villages in the Loyamorock ward were selected for the study implementation.

### Study design

The results presented in this paper are based on the qualitative arm of a mixed methods study. The study was conducted using a pre-intervention, intervention, and post-intervention study design. A total of Sixteen IDIs were conducted with CHEWs; 16 IDIs with Opinion Leaders, 8 semi-structured interviews with county, sub-county NTD program personnel and county trachoma focal person, 5 FGDs with CHVs, 16 FGDs with community members and 5 micronarrative surveys with trichiasis patients.

### Data collection

With the aid of interview guides, the data was collected using Pokot, which is the local language. Two social scientists from KEMRI moderated the data collection, assisted by 8 trained field assistants recruited from the community. The field assistants were trained on Trachoma, and this included; pathophysiology, symptoms, diagnosis, complications, prevention, treatment, and management. They were also trained on qualitative data collection methods which included; data collection techniques, proper use of voice recorders, and data transcription. Each FGD and each IDI were held in private areas to ensure participants’ confidentiality and to avoid interruption. Notes were taken during the data collection process and voice recorders were used to record all the information in the local *Pokomo* language.

### Data management

The data management process started with a collection of information gathered through field notes and audio recordings. The recorded data were coded and later transcribed and translated into English. To minimize bias, double transcription and translation and back translation were done among the investigators. The hard copies of the data were stored in lockable and secure cabinets and the soft copies were stored in password-protected computers. Access to the hard copies and transcripts was only upon authorization by the Principal Investigator.

### Data analysis

The coded data from the IDIs and FGDs were entered into QSR NVIVO version 10 software This technique enables the identification and exploration of themes within the coded data [9]. A code list was developed comprising broad themes, which were iteratively agreed upon among the research team members after a preliminary reading of the IDI and FGD transcripts and later modified to accommodate emergent themes. The textual data was coded into selected themes and a master sheet analysis was carried out, giving all the responses a theme. Further, manual analysis was conducted according to study themes, which were determined before the analyses. A thematic framework approach was adopted where responses were categorized into themes and then ideas formulated by looking at the patterns of responses [9]. The analyzed data were presented in text form. The coding framework is shown in Table 1.

**Table 1:** Thematic coding framework.

Contextual factors	Social & Environmental factors	Programme factors
Low community education and knowledge of trachoma cause, prevention, transmission, control	Cultural	Community sensitization, delays in receiving supplies
Side effects	Demographic	Transport
Risk perceptions	Socio-economic	Training of MDA
Awareness of MDA treatment benefits	Physical	MDA planning
Attitudes towards modern medicine	Security	Staff motivation and workload
Distribution method		Delays in drug delivery
Interaction with drug providers		Storage
Stigma		Schedule of MDA
		Distribution to the community distributors for MDA

Source: Authors Field Data, 2025.

## Results

### Background characteristics of the study participants

#### The county leaders.

Eight county and sub-county leaders in-depth interviews were conducted. The participants comprised the county trachoma focal person, the county NTD coordinator, the sub-county eye focal person, and the sub-county disease surveillance coordinator. A majority, 75% were male and their age range was 50–59 years.

#### The CHVs.

Four FGDs were conducted among 48 CHVs who had participated in the trachoma MDA program. The majority, 58.3% were female. All were Christians and had attained secondary education.

#### The opinion leaders.

Fourteen opinion leaders participated in the IDIs. A majority, 64%, were male, 57% were salaried workers, and their age range was 40–49. Only 21% had a level of secondary education or higher.

#### The community members.

A total of 16 FGDs were conducted among the community members. The majority, 51%, were female, and their age ranged between 19 and 49. Most of them, 68%, had their level of education up to secondary school, and 92% were farmers.

#### Micronarrative surveys.

Six micronarrative surveys were conducted among the trichiasis patients. The majority, 66% were female with 83% having an ager range of fifty years and above. Half of them (50%) did not have any formal education while the other half (50%) had a level of education up to primary school and the majority, 50%, were pastoralists (n = 3).

The socio-demographic characteristics of the study participants are presented in Tables 2 and 3.

**Table 2:** Socio-demographic characteristics of the Qualitative arm study participants.

	County leaders	Health workers	Opinion leaders	Micronarrative Survey
	Frequency (n = 8)	Frequency (n = 8)	Frequency (n = 14)	Frequency (n = 6)
Gender				
Male	6 (75%)	5 (62.5%)	9 (64.3%)	2 (33.3%)
Female	2 (25%)	3 (37.5%)	5 (35.7%)	4 (66.6%)
Age*				
20-29			2 (14.3%)	
30-39	1 (25%)		2 (14.3%)	1(16.66)
40-49			6 (42.8%)	
50-59	3 (75%)		4 (28.6%)	5 (83.3%)
≥ 60				
Marital status*				
Single				
Married	8 (100%)	4 (100%)	14 (100%)	4(66.66%)
Divorced				1(16.66%)
Widowed				1(16.66%)
Education*				
None			3 (21.4%)	3 (50%)
Never Completed Primary education			2 (14.3%)	
Primary education*			2 (14.3%)	3(50%)
Secondary education*			1 (7%)	
College	8(100%)	4 (100%)	2 (14.3%)	
Religion*				
Practicing	8(100%)		13 (92.86%)	3(50%)
Non-practicing			1 (7.14%)	3(50%)
Occupation				
Farmer			4 (28.57%)	
Business			1(7.14%)	
Salaried worker	8 (100%)	4 (100%)	8 (57.14%)	
Casual laborer				
Pastoralist			1 (7.14%)	3(50%)
Nurse				1(16.66%)
Unemployed				

Source: Authors Field Data, 2025.

* Some data missing.

* Includes people who received some education but may not have completed this level.

* Includes people who received some education but may not have completed this level.

**Table 3:** Socio-demographic characteristics of the study- FGDs participants.

Description	Frequency (N = 184)	Percentage (%)
Gender		
Male	81	44%
Female	94	51%
Age in years		
19-29	57	31%
30-39	59	32%
40-49	45	24%
50-59	22	12%
≥ 60	2	1%
Educational level		
None	15 32	26%
Never Completed Primary education	16	8.7%
Primary level	45	25%
Secondary level	64	35%
College	7	4%
Religion		
Practicing	172	93%
Non- practicing	12	7%
Occupation		
Farming	85	46%
Business	37	20%
None	47	25.5%
Pastoralist	9	5%
Salaried Worker	5	3%
Casual labourer	1	0.5%

Source: Authors Field Data, 2025.

### Barriers of community participation and access to mass drug administration for trachoma elimination

#### Contextual factors.

This theme presents the results of the contextual factors that affect MDA for trachoma and includes the following sub-themes: knowledge of trachoma, side effects of the drugs, risk perceptions, attitude towards modern medicine, drug distribution methods, awareness of MDA treatment benefits, attitudes towards modern medicine and stigma.

i Low community education and knowledge of trachoma cause, prevention, transmission, control

There is a basic understanding of trachoma, but there are misconceptions as it is attributed to flies, dust, dirt, and witchcraft. Other causes include mentions of “splits from trees” entering the eye. This is unlikely and could create confusion about the true cause of trachoma. There were references to allergic reactions to medication as a cause. There is an emphasis on poor hygiene, dirty environment, dust, unclean water, and not washing hands/face as causes of trachoma There is a lack of awareness about long-term complications, while most quotes mention blindness some responses don’t mention the irreversible nature of blindness. There are misconceptions about immediate impacts, responses associating trachoma with death, and the use of terms like “fool” and “poor,” may reflect a distorted view of the disease’s immediate impact. Discussions with women reveal concerns about typhoid, brucellosis, and sexually transmitted diseases (HIV) prominently as opposed to trachoma. Transmission is also observed to be via person-to-person.

ii Side effects

Several participants reported experiencing side effects like diarrhea, stomach upset, nausea, vomiting, and headache. Concerns about side effects being worse for pregnant women. The bitter taste and large size of the tablets can make them difficult to swallow, especially for children.

“The size of the drugs should be reduced, made small, as it too large for children.”

**FGD-AM-CM-002**

“It has a bad smell and taste that when I took the drug I vomited immediately After taking the drug. My eyes still ache, and I think it’s because I vomited all the drugs in my stomach. I have light sensitivity since then.”

**FGD-AW-CL-004**

iii Risk perceptions

There is a misunderstanding of the severity of trachoma in comparison to other diseases mentioned. This was observed as several diseases were mentioned more frequently than trachoma, including HIV, malaria, ulcers, and typhoid. There are allusions to old age and children as risk factors. Focus group discussions with both adult men and women suggest that the community feels men are less susceptible due to better hygiene habits. There is also a focus on children and the elderly being more susceptible to trachoma.

“…this disease affects mostly females, old age and children. It rarely affects men because men wake up and wash their faces in the morning than mothers and children. Old people are affected because they cannot help themselves in terms of self-cleanliness and environment. Men go bath with soaps, and this makes them free of the disease.”

iv Attitudes towards modern medicine

The majority of the participants who had suffered from the disease seemed unsure about MDA and the SAFE strategy. They practiced epilation of the eyelashes to alleviate pain and prevent blindness, caused by inward-facing lashes and were even assisted by family members. They expected their eyesight would recover following epilation and were disappointed when they did not observe any changes. community members still have more trust in traditional medicine as compared to modern medicine. Modern medicine, if sought, is only done so after traditional medicine fails.

“My grandchildren used to pull out the eyelashes in order to help relieve pain of the eyes. My grandchildren were disappointed of removing of the eyelashes because they did not see changes of it, am not seeing even after removing the eyelashes.”

IDI-MS-NY-003

“Some refused to take the drugs, they said it was for family planning”.

IDI-HW-004

“I decided to sell my livestock to go for further medication attention, where I visited a traditional medicine man who further instructed me that is a witch. They told me to bring a black goat to chase away the evils in my boma. I do all this, but it was in vain only to waste my resources.”

v Distribution

There is limited accessibility to remote areas such that, the distribution might not reach households, potentially excluding some community members, particularly children or those with mobility limitations. The participants have to travel long distances to access the drug distribution points. There is incomplete household coverage, concern that “receiving only one dose is not enough for the whole family” expresses concern that receiving only one dose is not enough for the whole family. This is because drugs are only administered to those present in the household or those who can go to the distribution centres. There are inconvenient distribution methods such as slow walking distribution across villages might be inefficient as highlighted by some community members. Lack of accessibility in remote areas, for instance, villages like Lokrakau. Short distribution periods may not allow everyone to participate, especially considering nomadic lifestyles. Issues with distributors such as bias of previous distributors and lack of familiarity with local languages can create distrust and hinder participation. Lack of community involvement shown by community responses of exclusion of village elders and youth from distribution and sensitization efforts can lead to a sense of detachment.

“…. for example, we can hear the drug distribution is like today and those who are living far away from the centers are not able to get the drug. Only those living near are the ones receiving the drugs.”

**FGD-AM-CL-001**

“Employing house to house method of drug distribution so that the drugs could reach even to all the community members and especially those who are the victims of Trachoma.’’

**IDI-OL-CM-001**

“The best season for the drug distribution to be done in this region is during the rainy season. During this period, all the people in the community will be present since they would have already migrated back with their livestock from other places where they went to look for green pastures and water during the drought season.”

**IDI-OL-CM-001**

“There were not enough teams in the last MDA exercise to cater for the Tiaty sub-county with dispensation of drugs and supervision. Transportation of the teams was an issue, the vehicles hired by the partner organization were not enough.”

**Eye focal person, Tiaty East Sub-County**

“The problem is that the drug distributors are few and more should be employed so that they can reach the drugs to the people living in the reserves and make the activity run successfully.”

**IDI-OL-CM-001**

“The current drug distributors should be added in large numbers. These large numbers help reach every corner of the area.”

**IDI-OL-CM-003**

“Language barrier. Drug distributors were speaking in Swahili language while community members never understood the message.”

**IDI-OL-CP-005**

vi Stigma

Stigma was a barrier to MDA as community members noted that trachoma is punishment for wrongdoing in society, not a disease that could be treated with drugs. Blindness caused by trachoma. affected their behaviour and caused physical impact, psychological distress due to stigma, and economic impact.

“It started 15 years ago as diarrhoea which later led to poor vision of my sight and after testing in the hospital, I was told this is trachoma. It ended up forcing me to live this miserable life that is full of dependency and failure in life, wonder where I was mistaken in this earth.”

**Participant 1_MS**

“Many of my things have become distracted even my family can’t gather their daily food properly. I can’t walk on my own due to this disease even my children I don’t know all of them.”

**“Participant 2_MS**

#### Social & environmental factors.

i Cultural

All the study participants noted that the cultural nomadic lifestyle in search of pasture affected the MDA uptake especially when carried out during the dry season. The community members migrated during the dry season, only to return to their homes during the rainy season, as there is enough pasture and water for their animals.

“The issue of migration especially due to drought is also another challenge, as the members of the community have to look for water and pasture for their livestock.”

**Eye focal person, Tiaty East Sub-County**

ii Physical

All the participants noted that access to MDA distribution points was hindered by poor roads and terrain, especially in the very remote areas and poor road network, which hindered communication between distribution teams. Flooding is common in the rainy season in Loyamorok ward.

“There is also a challenge in infrastructure. The roads and terrain are very poor.”

**Eye focal person, Tiaty East Sub-County**

“…poor network coverage which hinders timely communication through mobile phones.”

**Baringo County NTD Coordinator**

“….. And then also if at all it (MDA) happens during the rainy season, issue of flooding is something that might interfere with the program and this issue of movement during the drought season.”

**Sub-County Disease Surveillance Coordinator, Tiaty- East**

iii Security

Insecurity led to the MDA exercise being postponed in some areas affecting uptake. It was however noted that sometimes government security personnel escort the drug distributors.

“The challenges include insecurity in some areas making it unsafe. At times, the exercise has to be postponed when the area is inaccessible.”

**Eye focal person, Tiaty East Sub-County**

“In areas where there is fear of insecurity of course, we use the government security personnel who escort the drug distributors and provide Intel on the security of the area.”

**Baringo County Trachoma focal person**

#### Program factors.

i Community sensitization

A significant number of participants expressed a complete lack of prior information about MDA. Even those who had heard about the drugs lacked information about the program itself. There exist ineffective communication channels as community relies on young children returning from school or passive learning through overheard conversations. There is limited reach of current methods which focus on stationary locations like health facilities and markets that may miss those who are unable to travel. Mobile announcements using cars seem to have limited impact considering the lack of overall awareness. There is also the need to use posters to educate the community members in their local language.

“This disease is there but no one talks or teaches about it. Even those distributors of MDA do not reach us. We just have eye itch.”

**FGD-AM-CM-002**

“Only the literate people in this community knows more about the MDA than the illiterate who don’t know much about it.”

**IDI-OL-CP-006**

“The information given was too little they should have used big posters diagrams to educate the people in their local language, but am glad the information got people in the community which was a good thing.”

**IDI-OL-002**

ii MDA planning

Most of the community members noted that the number of people who are utilized in the information sharing process on MDA dates and other plans are very few, and thus are not able to cover all the villages in the ward. The majority of the opinion leaders noted that the time allocated for MDA sensitization, 2 days, was too short.

“There is not enough time given for the community to understand the information given because the sensitization was only done for 2 days and not all the people would have gotten and understood the full information at the end of those two days. This is because immediately after the sensitization day. The drugs were distributed on the next day.”

**IDI-OL-CM-001**

## Discussion

The belief that trachoma is caused by witchcraft could lead to resistance to medical interventions and a preference for traditional healing methods. Allergic reactions could lead to fear of side effects and hesitancy to take the medication. While hygiene is important for overall health, it may downplay the role of the bacteria and the effectiveness of medication. This focus could lead to individuals believing that improved hygiene alone will prevent trachoma, making them less likely to see the need for medication. Lack of knowledge on the irreversible nature of blindness suggests a need for education about the permanent vision loss caused by trachoma. This will ensure that community members seek treatment and participate fully in the MDA. If women are not involved in healthcare decisions, they may not be aware of trachoma or its treatment options. An incomplete understanding of transmission could lead to concerns about the medication itself causing the spread.

The mentioning of many other diseases as well as trachoma, suggests that trachoma may not be a top health concern for the community, potentially leading to lower participation in MDA campaigns. There is a need to develop and implement a targeted awareness campaign specifically focused on trachoma. This campaign should aim to educate the community about the severity and impact of trachoma as a prevalent health concern. While the demographics of old age may be more susceptible, they are not the cause. The misconception that men are less susceptible could lead men to prioritize other activities over participating in MDA. While the demographics of children and the elderly being more susceptible to trachoma may be more vulnerable, excluding others from the importance of MDA could decrease overall program effectiveness.

There is a need for education campaigns that address misconceptions and emphasize the bacterial cause and transmission routes of trachoma. Framing the message around the effectiveness of the medication in preventing blindness can increase its perceived value Additionally, emphasizing hygiene practices and the role of flies as transmission routes can indeed provide a tangible way to engage the community. Campaigns could showcase the importance of clean-living environments and encourage practices that reduce contact with flies, thereby making the message more relatable and actionable. Engaging community leaders and traditional healers can help dispel myths and promote trust in the MDA program. A targeted approach in education campaigns can effectively increase awareness, improve participation in prevention efforts, and ultimately reduce the burden of trachoma in the community. Promoting the benefits of MDA for the entire community can encourage wider participation. The likelihood of taking the offered treatment has been positively associated in several studies with the community’s knowledge and understanding of the disease, its transmission, MDA, and the community’s perceived risk of getting the disease [10,21–23]. A systematic review by Gammino et al. indicated that the major factors impacting health service uptake by nomadic pastoralists include distance/geographic access; limited knowledge among pastoralists on disease and awareness of health services pastoralist beliefs, behaviors, and attitudes toward formal health sector services [24]. Addressing logistical challenges like access to water, childcare, and transportation can improve participation, particularly for vulnerable groups. highlights the belief in witchcraft as a cause of trachoma. This could lead to seeking alternative treatments and distrust of government initiatives. Education campaigns should emphasize the irreversible blindness caused by trachoma and the importance of early intervention. Framing the message around the benefits of MDA in preventing blindness and improving overall health can increase program acceptance. Addressing misconceptions about the disease’s immediate impacts and causes can improve trust in the government’s program. Travel burden may potentially discourage participation, especially for the elderly or those with limited mobility. Participant concerns about receiving only one dose for the whole family suggest a need for clearer communication about how many tablets are needed per person and how to distribute them within households. Expanding distribution points to include community centres, schools, and markets can improve accessibility. Utilizing multiple methods like motorbikes, bicycles, and foot distribution by trained community health volunteers can ensure wider reach within villages. Providing clear instructions on household distribution during drug administration and education campaigns can address concerns about incomplete coverage. It’s important to note that some quotes also highlight positive aspects of the drug distribution process, such as the use of local languages by distributors and a sense of community cooperation. Building on these positive experiences can further strengthen the program. Lack of awareness highlights a critical gap in communication. The fact that even those who had heard about the drugs lacked information about the program itself suggests unclear messaging or insufficient education. These findings are consistent with the results of studies conducted in Kenya, Ghana, India, Indonesia, and Haiti [5,25,26] suggesting a lack of targeted communication strategies. A complete lack of awareness about MDA suggests unclear messaging or insufficient education. While some mentioned chiefs, village leaders, and barazas as information sources, the ineffectiveness is evident in the overall lack of awareness. Implement a multi-pronged communication strategy that utilizes various channels like community radio, mobile phone text messages, and social media groups to ensure wider reach. Train community health volunteers, religious leaders, and traditional healers to act as trusted sources of information. Organize educational campaigns in villages that explain trachoma, the benefits of MDA, and how to participate effectively. Based on the community responses the preferred communication channels included posters in the local language. Forming groups specifically for spreading awareness during campaigns suggests a desire for active community involvement. Utilizing youth groups demonstrates a preference for engaging younger demographics in spreading information. Involving Community Health Volunteers leverages their existing connection to the community. Holding community meetings (barazas) was suggested as a way to educate people about trachoma and the importance of MDA. Utilizing market days, where a large number of people gather, for awareness campaigns was seen as an effective strategy. Including schools in awareness campaigns highlights the importance of reaching children. Providing training sessions for community members before MDA can improve program understanding and participation. Conducting annual awareness campaigns emphasizes the ongoing fight against trachoma. Provide clear instructions on how to take the medication, including the importance of taking it with food. Develop age-appropriate formulations (like chewable tablets for children) to improve palatability and ease of administration. Conduct pharmacovigilance to monitor side effects and implement strategies to minimize them. Localized distribution points may not reach everyone, particularly in remote villages. Exclusion of village elders from distribution and sensitization efforts can lead to a sense of detachment. Implement a multi-pronged distribution strategy that includes house-to-house visits, distribution points at water points, churches, schools, and markets, extend the distribution period to accommodate nomadic communities and those who miss initial rounds. Train local community members, including CHVs and youth, to act as distributors to improve cultural sensitivity and accessibility. Partner with village elders for community mobilization and sensitization.

## Conclusion

This study explored potential barriers that could affect efforts in equitable implementation of MDA and ensure that no one is left behind from PC in pastoral and remote settings of Baringo County, Kenya. This study documents important challenges for consideration when implementing trachoma MDA campaign. This is useful especially during design and implementation, to make the program more efficient and achieve the goal of trachoma elimination. Given NTDs are targeted for elimination by 2030 as part of the sustainable development goals; implementation of MDA should consider the setting of the interventions particularly in pastoral and remote areas to reach all segments of the population. Further, our study findings may be useful in improving the implementation of MDA in other similar settings.
